# ‘If experts differ, what are we to do in the matter?’ The Medico-legal Investigation of Gunshot Wounds in a 1927 Scottish Murder Trial

**DOI:** 10.1093/shm/hkw066

**Published:** 2016-07-11

**Authors:** Nicholas Duvall

**Keywords:** forensic medicine, gunshot wounding, Edinburgh, Henry Harvey Littlejohn, John Glaister, Bernard Spilsbury

## Abstract

This article uses a notorious criminal trial, that of John Donald Merrett for the murder of his mother, as a case study to explore forensic medicine’s treatment of gunshot wounding in pre-war Scotland. This topic, which has hitherto received little attention from historians, provides insight into two issues facing the discipline at this time. First, the competing attempts by prosecution and defence expert witnesses to recreate the wound in a laboratory setting, in order to determine the distance from which the shot had been fired, exposed the uncertainties surrounding the application of a well-known laboratory technique for which no fully agreed-upon protocol existed. Secondly, the case allows the examination of the working relationship of a medical expert and a gunsmith, in which disciplinary boundaries became indistinct and the wound a shared site of analysis, in a period before the separate profession of forensic science became institutionally grounded in Scotland.

In February 1927, Edinburgh was gripped by a sensational murder trial. John Donald Merrett, a student, was accused of shooting Bertha Merrett, his mother, behind her right ear, after she discovered that he had been forging her signature on her cheques to fund a lifestyle of nightclubs, motorbikes and dancing instructresses. Mrs Merrett survived for two weeks in hospital, drifting in and out of consciousness, before dying on 1 April 1926 of meningitis, a complication of the wound she had received.^1^

It had not been a smooth road to prosecution. After interviewing her son, the police initially believed Mrs Merrett had attempted suicide. It was only in the summer, after the fraud was discovered, that a murder investigation commenced. In order to prove that the deceased had not shot herself, the Crown Office (Scotland’s prosecuting authority) commissioned a series of experiments from two university professors of forensic medicine, Henry Harvey Littlejohn from Edinburgh and Glasgow’s John Glaister. They fired the handgun recovered from the scene at a series of targets from various distances, measuring the extent of smoke and powder blackening deposited on each. Comparing the results with the appearance of her wound, they concluded that the shot had been fired from such a distance that Mrs Merrett could not have shot herself. Merrett was duly charged. However, his defence hired their own experts, the famous pathologist Bernard Spilsbury and the London gunsmith Robert Churchill. Their experiments showed the opposite: a self-inflicted wound was possible. An impasse followed.^^2^^

The case attracted considerable public interest, with extensive press coverage both in Scotland and further afield.^^3^^ The sensational and dramatic aspects of Mrs Merrett’s death later inspired books and, recently, television documentaries.^^4^^ However, the case is also of considerable use in understanding forensic medicine in 1920s Scotland. At this time, forensic medicine and the scientific investigation of crime were undergoing considerable development, through the use of more advanced laboratory techniques, for example to analyse bloodstains and categorize them by species and blood group.^^5^^ Yet, in both Scotland and England, specialist non-medical forensic science services, either attached to or independent of police forces, had yet to achieve the institutional footing which they would in England by the 1930s and in Scotland by the 1940s. Thus, much of the work associated with incidents such as shootings was performed by an alliance of forensic medical experts and those from disciplines such as gunmaking, whose everyday work was not linked to the investigation of crime.^^6^^

This article uses the particularly extensive court records from the case, contemporary textbooks and journal literature, and press reports pertaining to similar cases, to examine two major issues facing forensic medicine in this period. First, a clash of two sets of well-resourced experts exposed the uncertainties surrounding the application of a well-known laboratory technique to a medico-legal problem: serious disagreements existed over the precise method for carrying it out, despite its having been cited in textbooks for decades. Second, in the absence of police scientists specialising in ballistics, the examination of the weapon and the analysis of its effects fell to medico-legal experts and gunsmiths, whose close collaboration illustrates how knowledge and expertise could merge between two disciplines, rather than adhere to strict disciplinary boundaries.

After a first section outlining the general history of medico-legal approaches to the gunshot wound, the second section examines the impact of procedural uncertainties within forensic medicine upon the trial. The prosecution and defence each disputed the validity of the other’s experiments, arguing that their setups were inaccurate representations of the original incident which failed to take circumstantial conditions into account. The prosecution was criticised for failing to regulate moisture, and for ignoring the effects upon their results of the material from which their targets were made; the defence for using a different weapon and ammunition to those found in the Merrett home. Such critiques shared parallels with early responses to the appearance of latent fingerprint identification in court in the early twentieth century, which also criticised supposedly artificial test conditions.^^7^^ Yet, while fingerprint examination went on to become rigidly self-policed, with clear protocols to ensure consistent testimony between examiners, the trajectory of forensic medical expertise on gunshot wounding was different.^^8^^ Although the technique for determining the distance from the wound at which the shot had been fired had been cited in textbooks since 1866, by the time of the Merrett case, as an analysis of technical literature shows, the technique rested on uncertain foundations. Authors had not agreed upon a clear protocol for its execution. The courtroom exchanges during Merrett’s trial demonstrate that this uncertainty was manifested in competing claims to experimental authority, which ultimately could not be resolved, and which provided ammunition for the lawyers’ cross-examinations to undermine witnesses’ expertise.

The broadening of the range of techniques used in forensic medicine was followed by a greater degree of collaboration between its practitioners and other disciplines. In a number of high profile Scottish cases, such as the investigation of the murder of Helen Priestly in Aberdeen in 1934 and the Ruxton case of 1936, forensic medical experts collaborated closely with bacteriologists and anatomists, among others, to solve highly complex problems.^^9^^ Such relationships remained important as the police’s separate laboratory capabilities were expanded, which in Glasgow began in the early 1930s and accelerated in the 1940s. Though the police were soon able to perform non-medical analyses using their own facilities, as Crowther and White note, doctors still played a role in corroborating their findings.^^10^^ This article’s third section analyses an early manifestation of the collaborative relationship, examining two contrasting ways in which medico-legal specialists and gunmakers worked together on the Merrett case. While the prosecution experts maintained a fairly well defined boundary between the dominions of gunsmith and medical expert, the division between the testimonies of the defence’s gun expert, Churchill, and principal medical witness, Spilsbury, was more fluid. They occupied a shared space in which the gunsmith was able to comment on the appearance and behaviour of human tissue, and the doctor on the composition of ammunition and the functioning of weapons. Newspaper reports and archival sources from this period show that this overlapping of expertise was not unique to this case, and took place at a relatively elite level, involving both prosecution and defence witnesses. This observation builds upon two sets of historiography on medical expert-witnessing: the first concerns the struggles for authority over the dead body between medical specialties; the second the degree to which medical understandings of evidence from the body were negotiated with viewpoints from outside of the profession.

In his work on the English coroner’s inquest, Ian Burney has shown the rivalries which existed between different branches of medicine in the late-nineteenth and early-twentieth centuries centring on whether a pathologist’s special knowledge of dead bodies or an attending general practitioner’s experience of a deceased person’s medical history was better placed to determine the cause of a death.^^11^^ Yet, the Merrett case demonstrates that under other circumstances, non-specialist views could be welcomed into the dominion of forensic medicine to collaborate on the production of expert opinion. The exchange between disciplines worked in both directions. Gunsmiths gave evidence relating to wounds, and medical witnesses gave opinions on the composition of ammunition and the functioning of firearms. This bears similarities with the evolving relationship between clinical medicine and the scientific laboratory in Scotland at the turn of the twentieth century. Steve Sturdy has shown that there was then a mutually beneficial relationship between the laboratory of the Royal College of Physicians of Edinburgh and the local medical community. Doctors sent samples for analysis to the laboratory, whose findings assisted them in their diagnostic and treatment decisions. Likewise, evidence from these submitted cases helped the laboratory’s researchers advance their own work.^^12^^ This amalgamation of clinical and laboratory knowledge to solve problems, whether in the treatment of a patient or the understanding of a physiological phenomenon, mirrors that found between the disciplines in the Merrett case, as doctors and gunsmiths worked closely to interpret the appearance of a wound.

However, what occurred between the two disciplines in the Merrett case was more than just a close working relationship. Indeed, it resembles the interrelation identified by Victoria Bates between medical and lay understandings of the signs of venereal disease in cases of alleged child sexual abuse in nineteenth- and early twentieth-century England. When diagnosing diseases such as gonorrhoea in child patients, general practitioners, lacking expertise in bacteriological diagnoses or the facilities to perform them, relied, in part, upon parents’ observations of visual signs, such as vaginal discharge. Thus, lay understandings of venereal disease were brought to bear upon medical ones. The two viewpoints often coincided: ‘The idea that early gonorrhoea could be “attended by a yellowish-white discharge” was evidently not only widespread in medical literature, but also part of wider lay knowledge about the signs of “healthy” and “unhealthy” bodies.’ Bates suggests that this brought an element of negotiation to this diagnosis, as ‘doctors may have shared and discussed their naked-eye strategies for understanding and interpreting such discharges’ with parents.^^13^^ Although there are many differences between the contexts discussed by Bates and the Merrett trial (where the doctors were specialists in forensic medicine rather than general practitioners), the concept of a negotiated understanding between medical and non-medical viewpoints, producing a conclusion to be argued in court, is helpful when it comes to understanding the role of the defence experts in this case. This was manifested not only in the way in which Churchill and Spilsbury worked together to perform the crucial experiments to determine firing distance, but also by the defence’s calling upon Churchill, before and during the trial, to speak to medical matters, for example critiquing a report written by Glaister, or to comment on the behaviour of a bleeding wound. This analysis builds upon Bates’s by demonstrating that such combinations of medical and lay knowledge were not only a feature of under-resourced general practitioners’ encounters with the law, but part of forensic practice at a much more elite level, albeit contingent upon circumstances which required different disciplines to collaborate.

## Wound Investigations

Recent years have seen a growing body of scholarship on the history and sociology of forensic medicine and science. Studies have ranged from examinations of how particular disciplines and technologies, such as toxicology, fingerprinting and DNA profiling, were established and granted authority, to institutional histories and discussions of the status of the expert witness as a whole.^^14^^ However, little has been written on the history of the use of forensic medicine and science to investigate shootings and firearms wounding, even though it represented an important interface between medical and non-medical evidence, and has long attracted the attention of medico-legal authors. Thus, it is worthwhile giving a brief outline of the history of this area.

Examining a wound to gain information about the manner of a shooting was not new in 1926. Alfred Swaine Taylor, arguably the preeminent medico-legal expert of the Victorian era, wrote in his 1844 *Manual of Medical Jurisprudence* that the appearance of the wound could indicate the distance and direction from which the shot had been fired, which suggested whether it was self-inflicted or not. A wound with ‘blackened’ edges, ‘as if they had been burnt’, suggested a near discharge, due to the heat and flame from the burning gunpowder, while an ‘oval or vulvular’ wound was indicative of the projectile having struck at an oblique angle, details which might help to determine where the shooter had been standing.^^15^^ Further material on the assessment of wounds was added in later editions of the book. In the 1866 edition, Taylor cited experiments carried out in the 1830s by a French medical jurist, Lachèse, who fired at human cadavers from various distances, using different types of projectile, including bullets and lead shot, noting the changing wound characteristics.^^16^^ It is unclear, however, whether Taylor’s readers were expected to carry out similar experiments for their own cases.

Taking successive editions of Taylor’s textbook as points of reference, the next major expansion of knowledge of wounding took place around the time of the Second Boer War of 1899–1902. This was a consequence of the fierce fighting and the implementation of new weapons technologies. Just before its outbreak, in 1899, two doctors, Arthur Keith and Hugh Rigby, performed a systematic study of the destructive effects of a number of different kinds of projectile fired into a range of targets, from bars of soap to human skulls.^^17^^ As the war progressed, and the enormous scale of casualties grew, surgeons had many opportunities to make new observations about the behaviour of wounds.^^18^^

Although military rather than medico-legal, this work was cited in the 1905 edition of another of Taylor’s textbooks, *The Principles and Practice of Medical Jurisprudence*, by then edited by Frederick J. Smith, lecturer in medical jurisprudence at the London Hospital. This edition took a more scientifically informed approach than before, incorporating factors such as muzzle velocity and air pressure into explanations of wound appearance.^^19^^ Smith had also carried out some experiments of his own into the distances from which a shot would burn the wound or the victim’s clothing, which led him to the conclusion that general rules about this phenomenon could not be given. ‘The facts in any given case can only be determined with experiments with the actual weapon used, and loaded as nearly as possible in the same manner as it was when used for the purposes which are being investigated’, he wrote.^^20^^

Speaking in 1907, Littlejohn, who later gave evidence for the Crown in the Merrett trial, also laid emphasis on the importance of specific experiments:

General experiments were of little value. In any given case, before a definite conclusion could be arrived at, experiments must be made with the particular weapon, and under the same conditions as regards the charge of powder and shot.^^21^^

However, not all authorities were so strict on this matter. Littlejohn’s comments had been made after a presentation by the eminent London expert in forensic medicine William Willcox, lecturer in forensic medicine at St Mary’s Hospital and Senior Scientific Analyst to the Home Office. Qualified in both chemistry and medicine, he was best known as a toxicologist, making an important advancement in the application of the Marsh–Berzelius test for arsenic in 1912.^22^ However, he had other medico-legal interests besides poisons, and for his 1907 presentation he had performed a series of experiments to ‘furnish a valuable guide in deciding the actual distance at which a pistol was fired when the distance was near’, using a variety of types of revolver and ammunition, as well as different types of target, including ‘white cardboard, wash-leather [,] flannel on cardboard’ and ‘fresh human skin’.^^23^^ While he acknowledged that the appearance of a wound from a given distance would vary according to the weapon used, he did not insist on weapon-specific experiments as Smith and Littlejohn did:

As a preliminary, it must be insisted that with regard to any firearm wound, whether produced by a pistol, rifle, or shot-gun, no exact conclusions can be arrived at as to the distance from which the weapon was fired, unless the type of weapon is known, and also the nature of the cartridge or charge used.^^24^^

For him, it was enough to know the type of weapon used.

This ambiguity about the value of general rules continued into the 1920s. In his 1925 textbook *Forensic Medicine*, Sydney Smith, a professor at Cairo University who succeeded Littlejohn at Edinburgh in 1928, gave some general points about the different appearances of shotgun wounds inflicted from different distances, although he acknowledged their limitations: ‘These details are given merely as a working basis; they vary with each weapon and its charge, but they are the outcome of hundreds of experiments with different weapons, and therefore have a certain value.’^^25^^ However, he maintained that case-specific experiments were important: ‘If a weapon is found it must be experimented with in order to reproduce the condition found in the body.’^^26^^ Nevertheless, the text was ambiguous in other ways. First, these statements are only contained in the section on shotgun wounds. It was not made clear whether experimentation, if the weapon were available, was also necessary in cases involving handguns or rifles. Second, Smith neither provided clear instructions for carrying out an experiment into the distance from which a shot had been fired, nor specified which conditions had to be controlled, such as moisture or the type of target.

Willcox maintained that a general reference collection of targets, fired at with a variety of weapons from different distances, was valuable. Speaking in 1929 after a Medico-Legal Society presentation by Sydney Smith, he said of his collection, ‘They are very useful, because if one knows the weapon that is used and one refers to one’s patterns, if the wound has a series of patterns very similar you can say precisely the distance at which the missile was from the body.’^^27^^ While he did not mention the necessity or otherwise of specific experiments for every case (which would have been impossible if the weapon involved was not recovered), the fact that he advocated drawing precise conclusions from patterns produced with a different weapon put him at odds with Littlejohn’s position. Such a view could be seen to lend authority to the experiments of Churchill and Spilsbury in the Merrett case, most of which were performed with a different weapon to that used in the incident under investigation.

There was, therefore, a lack of consensus about the importance of carrying out experiments specific to the shooting under investigation. Willcox and Sydney Smith recognised the value of general principles for estimating the distance from its target at which a gunshot had been fired. Littlejohn and Frederick Smith did not. There was also a lack of a clear protocol for performing shooting experiments. These facts are significant when it comes to understanding the disputes which took place between the prosecution and defence in the Merrett case regarding the validity of each other’s experiments.

## Making Sense of the Wound

The effects of the lack of clarity surrounding the experimental replication of firearms wounds are clearly shown by the Merrett case. Two well-resourced, opposing views of how to conduct firing-distance experiments were brought to bear in one trial, for which a substantial body of source material survives, including a trial transcript (which was not created for every court case), pre-trial documents and newspaper reports. The lack of accepted means of performing these experiments made it difficult for either side to use their experimental conclusions to produce a convincing argument as to what had occurred in the sitting room of the Merrett home the previous March, and gave each side’s lawyers ammunition to discredit the other’s evidence.

Convincingly replicating Mrs Merrett’s wound was always going to be difficult. The challenges of disciplining the chaotic outside world in the context of the wound-research laboratory, quite apart from the adversarial courtroom, have been well discussed.^^28^^ There were, however, particular problems associated with the Merrett case. Proving the fraud was relatively straightforward. A handwriting expert and an engraver were able to show the court that Mrs Merrett’s signature had been forged on her cheques. However, there were no reliable witnesses to the shooting, and a number of investigative failures had been made in the incident’s aftermath. For example, the police constable who took possession of the pistol could not recall whether he had picked it up from the floor, from the writing bureau or whether Merrett had handed it to him.^^29^^ There is no evidence of fingerprints having been taken. The maid, Henrietta Sutherland, initially supported Merrett’s version of events (that his mother had shot herself while he had been in the room) by saying she had seen Mrs Merrett drop the gun. She later recanted. While in hospital, Mrs Merrett was officially under arrest, because attempted suicide was an offence. Despite periods of lucidity, her visitors were not allowed to discuss the incident with her. She did tell the doctor, Roy Holcombe, what she remembered of the incident: she had been writing a letter; she recalled her son having been beside her, then hearing an explosion. However, when Holcombe told this to the police, they did not attempt to speak to Mrs Merrett about it while they still could. No statement or deposition was ever taken from her.^^30^^ Medical and scientific evidence would thus be crucial if murder was to be proved.

The most significant medical challenge related to the wound itself. Its appearance immediately after the incident would have been of greatest interest to investigators. However, seeing the wound in this state was impossible by the time investigations began. First, Mrs Merrett lay in hospital for two weeks between being shot on 17 March and her death on 1 April. By the time of the autopsy, her wound had partially healed, and so its condition had changed. Second, when she was admitted to hospital, the injury would have been washed, possibly removing blackening from the wound. Thus, the condition of the wound had to be attested to verbally by the staff who had attended to her when she was admitted to the infirmary, since the wound does not appear to have been photographed when it was fresh.^^31^^ The defence suggested that the account of the wound by the doctors and nurses from the hospital was unreliable, or at least irrelevant, because agitation during transport to hospital could have caused blood to be dislodged from the wound (possibly taking some blackening with it) before it could be examined properly.^^32^^ Even if the experimental attempts to reproduce the wound were successful, comparing their results to the original would not be a simple matter.

Experiments were performed on behalf of the Crown by Littlejohn, who as Edinburgh police surgeon had performed the post-mortem examination of Mrs Merrett, and Glaister, his Glasgow counterpart. They were the two preeminent medical expert witnesses in Scotland at the time who, in other circumstances, could have found themselves called by opposite sides. There were several levels of forensic medical practice in Scotland at this time. While any doctor could be called to examine a body found dead under unexplained circumstances, more complicated work was often tasked to the departments of forensic medicine located in university medical schools, of which Edinburgh and Glasgow were the most prominent. Here, experts had access to laboratory equipment, and possessed much more specialised knowledge than general practitioners.^^33^^

The experiments were carried out within the Forensic Medicine Department at the University of Edinburgh over two dates, first by Littlejohn alone on 6 August 1926, and second, by both professors together, on 8 December.^^34^^ In order to measure the extent of powder blackening left on a target from various distances, shots were fired from the pistol that had killed Mrs Merrett at cardboard targets and an amputated limb.^^35^^ When a shot was fired, particles of gunpowder emerged from the barrel of the gun and were embedded in the target if at close quarters. The less of this blackening there was, the further away the shot had been fired, meaning it was more likely to have been fired by someone else. Textbooks such as Smith’s carried illustrations of blackening patterns so that doctors would know what to look for at autopsy, though the extent of blackening could vary according to the type of gunpowder in the cartridge.^^36^^ At the post-mortem, no blackening had been found on Mrs Merrett’s wound, nor had any been noticed by the medical staff who had attended her in the hospital.^^37^^ Initially, this had not been regarded as necessarily suspicious; Littlejohn’s report on the post-mortem had stated that ‘there was nothing to indicate the distance at which the discharge of the weapon took place, whether from a few inches or a greater distance’, and he had not ruled out suicide or accident.^^38^^ In the light of the forged signatures on the cheques, however, an absence of blackening, which had been regarded as inconclusive, was now suspicious, and indicative of a more distant discharge by someone other than Mrs Merrett: her son.

An alternative scenario was that blackening had been present initially, but had been washed off by bleeding and the cleaning of the wound in hospital, escaping the notice of the hospital staff. In order to check for this, Littlejohn and Glaister’s experiments included efforts to remove blackening from their test targets by rubbing them with a sponge. The marks remained in place.^^39^^ Thus, it was unlikely that there had been any blackening on Mrs Merrett’s wound to begin with; otherwise it would have survived the treatment. This did not bode well for Merrett.

However, this was not the end of the matter. Merrett’s lawyers mounted a robust defence around the scientific evidence. His advocate in court was Craigie Aitchison, one of Scotland’s most renowned and scientifically well-informed lawyers, who was notorious for his detailed dismemberments of expert testimony.^^40^^ It was not unusual for the defence to call their own expert witnesses in Scottish trials at the time (an Edinburgh expert might be called by the defence in a Glasgow trial, and vice versa), and in this case they commissioned their own experiments into the firing distance. The two most eminent Scottish expert witnesses were already working for the prosecution, so the defence called two London experts who had first worked alongside each other in 1913, Robert Churchill, a gunsmith who had previously advised the authorities on a number of shooting cases, and Bernard Spilsbury, a high-profile forensic pathologist famous for being a persuasive presence in the witness box.^^41^^ Their evidence differed substantially from Littlejohn and Glaister’s. In their tests, the defence witnesses found the powder and smoke markings left on the targets easy to remove. They therefore argued that there could initially have been blackening around Mrs Merrett’s wound, but which was then removed inadvertently in hospital. A self-inflicted shot at close range could not, therefore, be discounted.^^42^^

Presented with two contradictory versions of the same experiment, the debate in the courtroom centred upon which setup better replicated the conditions of the original shooting. This question reflected a major difficulty of using experimental evidence in court. In an early presentation of latent-fingerprint evidence at a 1911 trial in the United States, cited by Cole, a fingerprint examiner demonstrated his skill and the power of his technique by lifting and correctly identifying a print left by a juror on a pane of glass. The defence lawyer objected to this demonstration, because ‘the court was creating pristine experimental conditions unlike the messy state of the crime scene’.^^43^^ There was a fundamental gap between the experiment and real life that could not be bridged. As Tal Golan writes, with reference to early uses of chemical evidence in the 1820s, ‘The legal instinct … is to be suspicious of extrapolation from artificially created facts to the original events of the case and tends to demand that the experimental circumstances be as much as possible the same as those in the case at hand.’^^44^^

In the Merrett case, critiques of this sort were applied to both the prosecution and defence cases. The two sets of experts disagreed over which experimental conditions would affect the outcome, and so have to be controlled. The first such point of disagreement arose over the type of material used for targets. Although Littlejohn and Glaister had performed their experiments using targets made from both cardboard and human skin (obtained from the leg of an amputee after a recent railway accident^^45^^), they had only produced the cardboard targets in court. They considered these to be adequate. However, Churchill, the defence gunsmith, argued that targets made from card and paper were inadequate, because gunpowder and other particles from the shot would be more likely to adhere to them than to human skin. In his evidence, he stated that, during their experiments, he and Spilsbury had found it easier to remove blackening from skin than from paper or card. In particular, flake powder did not mark skin ‘to the extent that it indelibly marks paper’.^^46^^ Thus, the results of experiments using solely artificial targets would be distorted. When asked by Aitchison whether, in his view, ‘experiments upon paper carry you any length at all in a case of this kind’, Churchill answered that they did not.^^47^^ Aitchison put this point to Littlejohn, who stated that he had found both types of material to behave similarly. However, since the skin targets did not appear in the list of Crown productions, he was unable to show the matching results to the court.^^48^^

The two sides also differed on the effects of the moisture of the targets, and whether this should be regulated. Littlejohn and Glaister, for the Crown, had not attempted to do so. When cross-examined on whether his experimental conditions, regarding moisture and temperature ‘coincide[d] with the actual condition that prevailed at the time’, Littlejohn played down their importance: ‘You take a piece of skin, you fire at that, and you get a certain result. The temperature of the air and so on I think do not make any difference.’^^49^^ While he admitted that some of his skin targets had been moist, and some dry, he and Glaister had not addressed the issue of moisture variation systematically, and Littlejohn was not in a position to challenge the proposition put to him by Aitchison that ‘the degree of moisture in skin makes a very material difference to the degree of discoloration [by blackening]’.^^50^^ Spilsbury, however, was ready to attest to the impact of moisture. He stated that when paper targets were wetted, the powder was ‘deposited over a larger area’. Turning to the question of skin, he said that ‘the degree of moisture of the skin, or greasiness of the skin would no doubt affect the pattern produced at a given range’, which ‘emphasise[s] the difficulty of reproducing in experiments conditions that were actually present at the time’.^^51^^ This statement appears to have been speculative; Spilsbury did not state that moisture had been precisely controlled in the experiments he and Churchill conducted with skin targets. This might suggest that the defence’s interest in the effects of moisture was more about opportunism than scientific rigour. Advocates on both sides would of course focus on areas of potential uncertainty to which they could most effectively draw the jury’s attention. However, expedient or not, this source of uncertainty was a consequence of the vague definition of a technique in the textbook literature of a discipline whose *raison d’être* was the courtroom encounter.

The most serious difference between the prosecution and defence’s experimental method arose over the handgun and ammunition used by the defence in their crucial set of London experiments, in which the targets had been subjected to washing to demonstrate the removal of blackening. They had used a different brand of ammunition, and had not used the handgun found at the scene of the shooting. Thus, they failed to replicate the original incident in a supposedly crucial respect, contrary to Frederick Smith and Littlejohn’s advice. Spilsbury and Churchill were not unaware of this principle of experimental practice. Because they performed their main set of experiments in London, where they both lived and worked, they had not initially been able to use Merrett’s gun or the rest of the unspent ammunition found at Buckingham Terrace. They had, however, attempted to compensate for this. According to Spilsbury, they had obtained a pistol ‘having exactly the same length of barrel and the same bore’ as Merrett’s and cartridges ‘which corresponded as closely as possible to the description we received of those which were used in this case’.^^52^^ Spilsbury also said that when he got the opportunity to the compare London and Edinburgh cartridges, he found them to be ‘practically identical’.^^53^^ Churchill noted that the two sets of cartridges were both made by one manufacturer, Nobel. When examined by Aitchison, Churchill argued that differences between the cartridges were moot because the wound had been cleaned:

Aitchison: Although you got a difference in density between the Edinburgh and the London experiments, does it make any difference at all to the conclusion which you draw as to the probability of any blackening being removed if the wound were washed?

Churchill: No. As the wound was washed, it is impossible for me to determine any distance.^^54^^

Thus, in Spilsbury and Churchill’s professional judgements, their compromise had been acceptable.

Littlejohn took a very different view. When shown one of the defence’s targets by Aitchison, he protested that the London cartridges gave ‘a totally different appearance from cartridges such as were used on Sunday last [in Edinburgh]’, when Spilsbury and Churchill had visited Littlejohn’s laboratory, and finally had the chance to perform a few experiments with Merrett’s gun itself.^^55^^ The prosecution exploited this deviation from Littlejohn’s preferred method. Cross-examining Churchill, William Watson, the Lord Advocate (Scotland’s chief public prosecutor), forced him to agree that it was advisable to use ‘the actual weapon and with as identical powder and ammunition as you can get’.^^56^^ Under cross-examination, Spilsbury agreed that, in terms of ‘judging of the effect in the actual case’, the prosecution’s experiments, as well as the ones that the defence carried out in Edinburgh, with Merrett’s gun, prior to the trial, were to be preferred.^^57^^ Of these two sets, only the first, the prosecution’s, had used skin targets. The Lord Advocate took the point to its logical conclusion:

Watson: And if Professor Littlejohn found that the Edinburgh powder on skin could not be so easily washed away as in the case of your London experiments, again I ask you would you not prefer to take Professor Littlejohn’s experiments?

Spilsbury: No, I think a good deal depends on the degree and the extent of the rubbing, as well as on the condition of the skin at the time when the weapon was fired.

Watson: Assuming the conditions of rubbing and the conditions of skin being the same, you would agree that the Edinburgh experiments would be perfect?

Spilsbury: I think we ought to judge by the combined effects of both in such a case as that.^^58^^

Spilsbury refused to admit that the prosecution’s experiments were superior, raising another potential variable, the vigour with which the targets had been rubbed in the attempt to remove the blackening.

The Merrett case clearly shows the sources of uncertainty in the experimental replication of firearm wounds, including the characteristics of different target materials, the effect of moisture, and variation between different weapons and ammunition. The importance of these uncertainties was amplified by the adversarial system of courtroom enquiry, as well briefed cross-examining advocates, such as Aitchison, drew attention to weak points in the evidence. It is important not to dismiss the importance of the adversarial system in this regard: it was why evidential uncertainties mattered. Indeed, textbooks emphasised the importance of thoroughness when performing post-mortem examinations, lest an omission be used to trip up an expert in court.^^59^^ The discipline was built around the courtroom encounter, and hostile questions were expected. Thus, technical uncertainties presented a problem.

Some of the doubts about the evidence in the Merrett case are attributable to lawyerly flair and technique. This was particularly true of the question of the effect of moisture, the importance or otherwise of which was not discussed in the literature before or after the trial, but which was deployed by the defence. Also, Littlejohn was discomfited when Aitchison quoted a passage from Sydney Smith’s 1925 textbook, stating that an absence of blackening in suicides was relatively common when rounds containing smokeless powder were used, challenging Littlejohn’s position.^^60^^ However, some objections were based on experimental findings: Churchill and Spilsbury had found powder to be more adhesive to paper than skin. While this might appear all too convenient to the defence case, conversations reported by Churchill’s biographer suggest that uncertainty over the target material went deeper than what was reported in court. Churchill had apparently objected to the use of an amputated limb in his and Spilsbury’s experiments, because dead tissue behaved differently from living tissue, but his colleague overruled him.^^61^^ Such uncertainties, coupled with skilled advocacy, meant that two very different interpretations of the same procedure could be manifested in one case. As the eventual outcome demonstrated, the court struggled to resolve this impasse.

## Doctors and Gunsmiths—Shared Expertise

In addition to the differences in experimental technique exhibited by the prosecution and defence experts, the ways in which the two disciplinary groups on each side worked together were also distinct. The gunsmith employed by the Crown was restricted to giving evidence about Merrett’s weapon and ammunition, while his medical counterparts dealt with the wound, including the experiments to reproduce it. Conversely, the gunsmith and doctor hired by Merrett’s defence collaborated closely on the experiments, both giving attention to the wound. The case therefore provides an opportunity to examine how the relationship between medical and non-medical witnesses was manifested, at a time when a more diverse range of expertise was being brought to bear upon major criminal investigations. The boundaries between the two disciplines of gunmaking and forensic medicine were hard to define. Churchill and Spilsbury both testified on ostensibly medical and non-medical matters, occupying a shared disciplinary space. I will also use examples of other cases to demonstrate that, although in the Merrett case this phenomenon was restricted to the defence witnesses, it could also be true of prosecution witnesses.

In order to understand how this confluence of disciplines came about, it is first necessary to note the context of expert authority in the Scottish courtroom in this period. In the 1920s, the specialist, separate discipline of forensic science did not exist in Scotland. The police’s capabilities in this regard were minimal. For example, in Glasgow, police had to call upon the Metropolitan Police for fingerprinting services.^^62^^ The bulk of any necessary laboratory work, for example determining the origin of bloodstains, fell to university medical experts. When further expert knowledge was required, doctors, or the prosecuting authorities, could also call on representatives of other disciplines. In shooting cases, as will be shown below, doctors valued the knowledge of gunsmiths, even though their trade was unconnected to criminal justice. The legal system was willing to accommodate this, and criteria for expert status in Scottish courts were not defined in great detail. While Crown Office rules stipulated that a post-mortem examination must be carried out by ‘a qualified medical practitioner’, for other tasks (aside from analysis in poisoning cases, which had to be performed by one ‘familiar with chemical researches’) no specific qualifications were required.^^63^^ However, whenever a ‘medical or other report’ was obtained, the ‘names and designations’ of the authors were to be carefully recorded, suggesting that credentials were important, if not mandatory.^^64^^

The importance of qualifications and experience for attaining expert status is shown in the opening sections of courtroom testimony. At Merrett’s trial, each of the expert witnesses was introduced with reference to his occupation and, if appropriate, qualifications. For example, Littlejohn listed his degree, fellowship of the Royal College of Surgeons of Edinburgh and professorial chair in forensic medicine.^^65^^ As well as his lectureships, Spilsbury, the medical witness for the defence, was stated to be Honorary Pathologist to the Home Office and to have ‘a very large experience, extending now to a period of twenty years in the investigation of crime on its medico-legal side’.^^66^^ Churchill, the gunsmith, did not list any qualifications, but gave an account of his experience, namely that he had been ‘frequently called to give expert evidence by the Public Prosecutor in England’ and ‘on some occasions’ to see gunshot wounds at Charing Cross Hospital, London.^^67^^ His expert status was not challenged directly and it was never suggested that Churchill was unqualified to give evidence, or speaking beyond his professional competence.

The professional literature of forensic medicine from this period shows that the expertise of the gunmaker was recognised and valued. This was not just the case in Scotland. They also played a role in English cases in the period before regional forensic science laboratories began to be built in the mid-1930s.^^68^^ At a meeting of the Medico-Legal Society in 1923, William Willcox asserted ‘the importance in cases of revolver wounding of obtaining the opinion of an expert gunsmith with reference to the type of bullet, cartridge and fire-arm used’.^^69^^ On another occasion, he emphasised their considerable knowledge of firearms, ‘which the ordinary medical man would not be acquainted with’.^^70^^ Douglas Kerr, Littlejohn’s successor as Police Surgeon to the City of Edinburgh, also noted the authority of the gunsmith in the examination of weapons and ammunition, although he believed this task could also be performed by police officers.^^71^^ The gunsmith’s expertise was also recognised by non-medical authors. Alfred Lucas, a Cairo forensic chemist, wrote that matters ‘concerning the spread of shot and the distance and direction from which a firearm has been fired should be left to sportsmen or gunsmiths’.^^72^^

While these forensic experts acknowledged gunsmiths’ place in the investigation of firearms cases, they placed tacit limits on gunsmiths’ responsibilities, although the authors varied on where these limits lay. Willcox, and, ten years later, Kerr, both conceived the gunsmith’s role to be examining firearms and bullets. They did not envisage a gunsmith’s involvement in the examination of wounds, or the determination of the distance between the gun and its target. Lucas, conversely, considered that determining the firing distance was the job of the gunsmith, or sportsman, although he did not associate this with wounding. For him, the examination of weapons and ammunition was the job of the chemist, who could analyse the composition of propellants and projectiles.

Although opinions differed as to the precise role of gunsmiths in these investigations, doctors’ involvement was more controversial, at least for non-medical authors. Lucas was adamant that the testing of firearms should be left to chemists. Those who thought it straightforward enough for ‘any doctor or hospital assistant’ to perform were wrong.^^73^^ In the introduction to his 1921 textbook, he implied disapproval of medical encroachment into forensic chemistry: ‘Some of the subjects dealt with will be found described in books on Forensic Medicine. This, however, does not mean that these subjects are medical, for such is not the case.’ Conversely, he had been ‘careful not to encroach upon the medical side of any subject, but [had] limited himself strictly to the chemical and general aspects’.^^74^^ During this period in Egypt, where Lucas worked, at least one prominent medical expert did indeed rival chemists’ authority over the examination of weapons. Prompted by a spate of political assassinations, Sydney Smith carried out research on the identification of cartridge cases, based on the unique markings left on them by guns’ firing mechanisms.^^75^^ He gave the analysis of cartridge cases and projectiles its own section in his textbook.^^76^^ A decade later, Dr B. Kraft, of the Prussian Institute of Foodstuffs, Drugs, and Forensic Chemistry, wrote, in a similar vein to Lucas, that ‘except in the case of body wounds, ballistics has nothing to do with medicine. … I do not consider it wise for anybody to carry on ballistic investigations incidental only to other researches.’^^77^^ It is important to note, however, that these authors were not writing from a British perspective. Non-medical ‘police science’ was in a much more advanced state in Egypt and Germany, where it had an institutional base in state-run laboratories and its own authority to uphold, than in Scotland or England.^^78^^

Although there was controversy in the international literature over doctors crossing into the developing discipline of police science, the role of the gunsmith, envisaged by the British literature, was quite straightforward, being the examination of weapons and ammunition. This was reflected in the Merrett case by the division of labour between the prosecution’s medical witnesses and their gunsmith, Alan MacNaughton. While Glaister and Littlejohn concerned themselves with the wound, and the associated experiments, MacNaughton was confined to an examination of the weapon and cartridges. He testified that the gun was unlikely to be accidentally discharged easily, and that it was intended for self-defence. Its aim was too poor for hunting rabbits, Merrett’s stated reason for buying it. On the crucial question of the markings on a wound left by a near discharge, he agreed that with such a weapon blackening caused by the heat of the barrel and unconsumed powder would only be likely at a short distance, but he was unwilling to suggest ‘exact measurements in which the blackening might appear’ because he had not carried out his own experiments.^^79^^

The limited role of MacNaughton contrasts with that of Churchill, the defence gunsmith. As well as taking part in experiments, his testimony extended beyond the normal boundaries of gunmaking suggested by the scientific literature. He did address the areas covered by MacNaughton, albeit coming to different conclusions. He was much more willing to entertain the theory of an accidental discharge, noting that, of pistols of such cheap manufacture, ‘there is no reliance to be placed upon them’.^^80^^ However, Churchill also gave his opinion on the wound itself, ostensibly a medical matter. This was in line with his statement at the beginning of his evidence that he had previously examined gunshot wounds at Charing Cross Hospital.

The most obvious way in which Churchill entered medical territory in the Merrett case was when Aitchison, the defence advocate, examined him. He was invited into a speculative discussion of the behaviour of the wound, and the probable effects of the care Bertha Merrett had received when admitted to hospital. Aitchison asked him:

… to assume that the wound bled for a considerable time, that the wound became surrounded with blood, that right over the wound there was congealed or coagulated blood, and that a wet swab had to be applied by the surgeon who dressed the wound, with considerable pressure, to remove the blood. Assuming these conditions, would you expect to find any blackening at all?^^81^^

The language used by Aitchison, including words and phrases such as ‘wound’, ‘swab’ and ‘coagulated blood’, evokes images which seem to belong to the medical context. By asking Churchill to extend his conclusions beyond the experiments performed with Spilsbury to include the bleeding wound, Aitchison stretched the gunmaker’s remit into the medical sphere. Churchill’s answer was conclusive: ‘With these conditions it would be impossible to determine any blackness.’^^82^^ His confidence suggests familiarity with wounds and the effects of blood.

Churchill’s expertise was also called upon during the defence’s preparation for the case. A record of his and Spilsbury’s pre-trial discussions with Merrett’s solicitors survives among the Crown papers relating to the case. In Churchill’s statement, he offered a critique of Glaister’s report. Two of these criticisms concerned the interpretation of the gunshot wound. Glaister had taken Dr Holcombe’s description of it (‘there being little destruction of the tissue at the site of the entrance’) to mean the gun had been fired from a distance. Churchill disagreed, arguing, ‘a very close shot would cause a little destruction of tissue, whereas a distant wound is unaffected by the powder gases’, interpreting Holcombe’s description as ‘a little destruction’ rather than none. He also suggested that Glaister was misreading the wound regarding the direction of the shot: ‘The direction of the wound should not be taken in its entirety—its earlier course is the true direction and as I understand that this bullet bears marks or an indentation on one side, this is some evidence of a deflection.’^^83^^ Here, Churchill brought his knowledge of projectiles to bear upon the wound.

Churchill’s comments on Glaister’s report are significant not just for their content, which shows his confidence in thinking about wounding, but because of their context. His being asked to comment on Glaister’s findings shows that Churchill was considered competent to speak to a medical report. This and the above exchange with Aitchison on the effects of bleeding show the important role that the lawyers building Merrett’s defence played in blurring the boundary between the gunsmith and the medical expert. It was they who, in asking Churchill about wounds, or soliciting his opinion on Glaister’s report, ensured that a ‘lay’ view was brought to bear on a medical matter.

Although the state of the wound was a medical question, Churchill’s interventions were not irrelevant to his own discipline. For instance, a dent in a bullet was very much within his sphere. His testimony is thus more suggestive of a shared space between forensic medicine and firearms expertise, in which two sets of knowledge influenced each other. Such a proposition is supported by the fact that the medical witnesses on both sides themselves covered a broad area, encroaching into the provinces of the gunsmith and, to a degree, the chemical analyst. Of course, in this period before forensic science in Scotland (and England) gained an institutional base, these doctors had few options besides interesting themselves in the examination of the weapon. Littlejohn brought knowledge of the force required to fire the weapon to bear upon the question of whether it had been discharged by accident; his opinion was that this was unlikely because the trigger required a heavy pull of 5 pounds.^^84^^ This echoed an earlier statement of MacNaughton, whose testing found the pull of the trigger to be 5 pounds and 9 ounces, ‘a fairly heavy pull for such a small pistol’, suggesting a sharing of information between the two men.^^85^^

Spilsbury, the medical witness for the defence, strayed more explicitly into non-medical territory. For example, he stated that he had tested the Edinburgh and London powder grains, in order, presumably, to demonstrate the applicability of his own tests. He produced a vial of powder from one of the cartridges from the London experiments:

A portion of the powder removed from one of the cartridges is shown in this bottle in which the scales of a steel grey colour can be seen, rectangular, and the same size taken from the cartridges we used in London. I have since compared these with the contents of one of the cartridges in the experiments here [in Edinburgh], and I found that they are practically identical.^^86^^

When Churchill was also asked about the powder, his answer was similar to Spilsbury’s, but carried more caveats:

The powder is apparently similar, but gives different results. The London ammunition gives more tattooing, and the Edinburgh ammunition gives more smoke blackening.^^87^^

The non-medical character of the examination of powder is also suggested by the prosecution’s division of labour. It was the gunsmith, MacNaughton, rather than either of the doctors, who analysed the powder, ascertaining that each cartridge found in Merrett’s house contained ‘exactly similar’ smokeless powder.^^88^^

There are several possible reasons why disciplinary boundaries were more permeable on the defence side than on the prosecution in this case. Their university positions may have given Littlejohn and Glaister access to better facilities than Spilsbury, allowing them to experiment without the assistance of a gunsmith, while Spilsbury needed Churchill’s help with this. Churchill’s previous experience of wounds, likely more extensive than other gunsmiths, may have bolstered Merrett’s lawyers’ confidence in his competence in this matter. The different burdens of proof on prosecution and defence evidence (the latter only having to cast reasonable doubt on the former’s case) may have allowed defence witnesses more latitude. Yet, it is difficult to draw firm conclusions about what conditions facilitated interdisciplinary expert witnessing of this kind, because the circumstances under which it occurred varied greatly, as shown by newspaper accounts of other cases. For example, defence medical witnesses’ purviews could be extended without the aid of a gunsmith. In other cases, a convergence of medical and gunmaking knowledge occurred among prosecution witnesses: the phenomenon was thus not restricted to defence experts.

The extension of a medical witness’s role can be seen in the case of a fatal shooting in Renfrewshire in 1931. Here, as with the Merrett case, the prosecution and defence’s approaches differed. On the Crown side, while a Glasgow gunsmith’s salesman, A. A. Bryson, gave evidence about tests he had performed to ascertain whether cartridges found by police had been discharged from a particular weapon, the wounds remained the domain of the two Glasgow forensic medical specialists, Glaister and Frank Martin. They discovered one scorched central wound and 27 smaller wounds surrounding it, and argued that the shot had been fired from a short distance, 9–12 feet, from the victim. The sole defence witness, Sydney Smith, by this time a professor at Edinburgh University, had a broader purview. He commented both on the wounds and the probable direction of fire, this latter point having been derived from markings on the victim’s walking stick.^^89^^ Smith’s previous practice and research in Egypt had given him particularly detailed knowledge of firearms and their effects. Other cases from this period show that determining the position of a shooter from damage to objects found at the scene of an incident was not necessarily a question for doctors. That same year, in England, a gunsmith told a Dorset inquest how he had examined boards containing shot recovered from the scene of a shooting to calculate the distance from which a fatal shot had been fired and the direction of the projectile’s flight.^^90^^

Other Scottish cases demonstrate that forms of cross-disciplinary exchange present in the Merrett case could also occur among prosecution experts, for example through gunsmiths referring to wounding in their evidence. In the 1935 murder trial of John M’Guigan, Glasgow gunsmith A. E. Martin’s opinion on the distance from which the shot had been fired was derived from the appearance of the wound:

From the appearance of the shot as it took effect as disclosed in photographs [Martin] had formed the opinion that the shot struck Kerrigan from an oblique angle from the right. It had been fired from a point at the front of Kerrigan’s right shoulder, more from the side than the front. He carried out certain tests to try to arrive at an approximate figure of the range at which the shot had been fired which entered Kerrigan’s body. His conclusion was that the gun must have been fired from close range, not farther than eight yards.^^91^^

Although the word ‘wound’ does not appear in this newspaper account, the phrase ‘the appearance of the shot as it took effect’ means that it was the wound to which the gunsmith referred, albeit indirectly via a photograph. A Glasgow pathologist, John Anderson, who regularly appeared as an expert witness, gave his view on the same question. He also thought that the gun had been fired no more than eight yards from the victim. Asked by the judge, ‘You substantially agree, then, with the view expressed by the gunsmith?’ he affirmed that he did.^^92^^ Thus, a medico-legal specialist and a gunsmith were both considered experts in deriving the firing distance from a wound, the latter so much so that he was able to partly base his opinion on a photograph. In a 1941 case, a medico-legal specialist and a gunsmith co-wrote a report for the prosecution on the firing distance experiments they had carried out.^^93^^ Again, this was a medically oriented task, involving the interpretation of a wound, albeit requiring substantial knowledge of firearms. Practitioners from the two separate disciplines had co-written the report, and were therefore equally responsible for its contents, demonstrating the shared, cross-disciplinary aspect of the gunshot wound.

These cases from the years following Merrett’s trial show that medical experts and gunsmiths continued to work closely in Scottish shooting investigations. Although it was the English expert witnesses who displayed this trait most clearly in the Merrett case, close cooperation with other disciplines was becoming an ever more important feature of Scottish forensic medicine in this period. It was championed in particular by Littlejohn and Glaister’s successors to their university chairs, Sydney Smith and John Glaister Jr. While medical experts needed to be aware of ‘advances in every department of science’, successful investigations featured a ‘close relationship’ between the doctor performing the autopsy, laboratory workers, firearms experts and the police.^^94^^ This spirit of cooperation on the part of forensic medicine played an important part in the development of forensic science as a discipline within police forces in Scotland in the 1930s and 1940s. By the 1940s, as the capabilities of their laboratories had increased, it was largely police scientists, rather than gunsmiths, who analysed weapons and projectiles. Yet, medical experts were still called upon to corroborate these findings. This was important, Crowther and White suggest, because of doctors’ independent status: they could attest to the validity of work done in the police laboratory which might otherwise have been disdained, since it had been produced from within the police establishment, and might have been considered partial.^^95^^

## Conclusions

The study of this well documented Scottish murder trial, in which medical and experimental evidence was crucial, provides an example of the challenges of using laboratory experiments to analyse an incident from the outside world. Recreating the shooting of Mrs Merrett in a controlled setting, in order to determine the distance from which she had been shot, proved problematic because the technique to be used was vaguely defined. Unlike the technology of fingerprint examination, which matured to become governed by a set of strictly enforced protocols to ensure concurrence and uniformity of practice among its practitioners, the experimental reproduction of gunshot wounds, referenced in textbooks since the 1860s, lacked a clear standard operating procedure.^^96^^ This was shown in the Merrett case, as experts disagreed over the control of moisture, the importance of the target material, and whether it was necessary to use the specific weapon and ammunition found at the scene in every experiment, or whether approximate matches were sufficient. Two rival interpretations of the technique, with two very different conclusions, followed.

An explanation of the divergence of opinion in this case must acknowledge the context of the legal system and the adversarial trial. For instance, the significance of the defence experts’ *de facto* role of casting doubt on the prosecution’s scientific case in relation to the findings of their experiments cannot be ignored. Circumstances were significant, as demonstrated by the changing interpretation of Mrs Merrett’s blackening-free wound when evidence of her son’s fraud emerged. Likewise, skilled lawyers amplified any procedural discrepancies in order to attack their opposite number’s case and bolster their own. When interpreting these circumstantial factors in relation to the procedural uncertainties presented by the literature, it is most helpful to understand them as working in concert, rather than as alternative explanations for the impasse which arose between the experts’ rival results. Hostile cross-examination and procedural uncertainty were bound together. It was the exploitation of the latter which made the lawyers’ courtroom strategies effective; and it was the threat of eviscerating questioning which caused medico-legal authors, normally, to emphasise the importance of thoroughness. Regarding experts’ motives for finding particular results, it is not possible, with the evidence available, to determine whether, for example, Spilsbury and Churchill’s results were genuine or deliberately contrived. However, either explanation would have been facilitated by the confusion surrounding the technique at issue, since the absence of a universally accepted protocol allowed variations in practice and hence results. The Merrett case therefore provides an example of the mechanism by which circumstantial factors exerted an influence over medical evidence in court.

The case also illustrates how medical experts in this period worked with witnesses from another discipline to explain how a gunshot wound was produced. The absence of a professional group of non-medical forensic scientists in Scotland (and England) meant that the medical witnesses worked alongside gunsmiths, who were not involved in criminal justice in their everyday work. Although a relatively clear boundary was maintained between Littlejohn and Glaister’s evidence and the gunsmith MacNaugton’s, the relationship between Spilsbury and Churchill, for the defence, was much closer. Their disciplines’ demarcations became indistinct, and they shared the task of interpreting the wound. Churchill was permitted to speak to apparently medical questions, and during preparation for the trial, was considered competent to criticise one of the prosecution’s medical reports. While the degree of close interdisciplinary collaboration was greater on the defence side in this case, reports of later Scottish cases show that prosecution experts also participated in interdisciplinary exchange.

The Merrett case demonstrates that medicine’s negotiation with other disciplines over legal questions took place at an elite level, as well as among the general practitioners cited by Victoria Bates. As with the instances she records, in which doctors’ lack of access to resources or knowledge of bacteriology led them to draw upon lay understandings in the diagnosis of venereal disease, so the medico-legal experts in shooting cases’ links to gunsmiths’ knowledge were shaped by the contexts in which they operated.^^97^^ The absence of a cadre of specialist police scientists led them to form links with trades unconnected to the justice system. Nevertheless, cooperation between forensic medicine and others did not end when the police in Scotland expanded their own scientific capabilities. For example, by the end of the Second World War, even though, in shooting cases, the Glasgow police laboratory was performing its own tests to determine the distance from which a shot had been fired, medical experts from the university still played a role in checking and corroborating their findings.^^98^^

In the end, Merrett was convicted of ‘uttering’ for forging his mother’s signature on her cheques, and sentenced to a year’s imprisonment, but the jury acquitted him of the murder charge with an equivocal verdict of ‘not proven’. This proved controversial with hindsight. In 1954, having assumed the alias ‘Ronald Chesney’, Merrett was found dead after killing his wife and mother-in-law. In his memoir, Sydney Smith, a staunch critic of Spilsbury’s working methods, was clear about where the blame lay: ‘The slackness of the police and the credit given to the misleading evidence of Spilsbury and Churchill, who had made a mistake and were too stubborn to admit it, allowed Merrett to live—and to kill again. A worthless life was saved, and two innocent women were thereby condemned to die.’^^99^^

## Footnotes

1 William Roughead, ed., *Trial of John Donald Merrett* (Edinburgh: William Hodge, 1929), 5–12.

2
*Ibid*., 19–45.

3 ‘Surprises at Trial of Edinburgh Student’, *Sunday Mail*, 6 February 1927, 2; ‘Son and Mother: Trial of John Merrett’, *Sydney Morning Herald*, 9 February 1927, 15.

4 Tom Tullett, *Portrait of a Bad Man* (London: Evans Brothers, 1956); ‘All That Lies Behind Me—Sgeulachd Donald Merrett’, BBC Alba, 15 May 2014; ‘Scotland’s Murder Mysteries’, STV, 11 June 2015.

5 G. Roche Lynch, ‘The Medico-Legal Importance of Blood Grouping’, *Transactions of the Medico-Legal Society*, 1923–24, *18*, 65–75; Douglas J. A. Kerr, ‘Forensic Examination of Blood and Blood Stains’ (unpublished MD thesis, University of Edinburgh, 1927).

6 Norman Ambage and Michael Clark, ‘Unbuilt Bloomsbury: Medico-legal Institutes and Forensic Science Laboratories in England between the Wars’, in Michael Clark and Catherine Crawford, eds, *Legal Medicine in History* (Cambridge: Cambridge University Press, 1994), 293–313 298; M. Anne Crowther and Brenda M. White, *On Soul and Conscience: The Medical Expert and Crime:. 150 Years of Forensic Medicine in Glasgow* (Aberdeen: Aberdeen University Press, 1988), 82–113.

7 Simon A. Cole, *Suspect Identities: A History of Fingerprinting and Criminal Identification* (Cambridge, MA: Harvard University Press, 2001), 183–4.

8 Simon A. Cole, ‘Witnessing Identification: Latent Fingerprinting Evidence and Expert Knowledge’, *Social Studies of Science*, 1998, *28*, 687–712.

9 John G. Wilson, ed., *The Trial of Jeannie Donald* (Edinburgh: Hodge, 1953); John Glaister, Jr. and James Couper Brash, *Medico-Legal Aspects of the Ruxton Case* (Edinburgh: E. & S. Livingstone, 1937).

10 Crowther and White, *On Soul and Conscience*, 84, 94–5.

11 Ian A. Burney, *Bodies of Evidence: Medicine and the Politics of the English Inquest, 1830–1926* (Baltimore, MD: Johns Hopkins University Press, 2000), 107–36.

12 Steve Sturdy, ‘Knowing Cases: Biomedicine in Edinburgh, 1887–1920’, *Social Studies of Science*, 2007, *37*, 659–89.

13 Victoria Bates, ‘“So Far as I Can Define without a Microscopical Examination”: Venereal Disease Diagnosis in English Courts, 1850–1914’, *Social History of Medicine*, 2013, *26*, 38–55, 46.

14 José Ramón Bertomeu-Sanchez and Agustí Nieto-Galan, eds, *Chemistry, Medicine, and Crime: Mateu J.B. Orfila (1787–1853) and His Times* (Sagamore Beach, MA: Science History Publications, 2006); Ian A. Burney, *Poison, Detection, and the Victorian Imaginaton* (Manchester: Manchester University Press, 2006); Cole, *Suspect Identities*; Michael Lynch *et al*., *Truth Machine: The Contentious History of DNA Fingerprinting* (Chicago, IL: University of Chicago Press, 2008); Crowther and White, *On Soul and Conscience*; Tal Golan, *Laws of Men and Laws of Nature* (Cambridge, MA: Harvard University Press, 2004).

15 Alfred Swaine Taylor, *A Manual of Medical Jurisprudence*, 1st edn (London: J. Churchill, 1844), 409–10.

16 Alfred Swaine Taylor, *A Manual of Medical Jurisprudence*, 8th edn (London: J. Churchill 1866), 292.

17 Arthur Keith and Hugh M. Rigby, ‘Modern Military Bullets: A Study of Their Destructive Effects’, *The Lancet*, 1899, *154*, 1499–507.

18 Alfred Swaine Taylor, *The Principles and Practice of Medical Jurisprudence*, 5th edn, vol. 1 (London: J. & A. Churchill, 1905), 552.

19
*Ibid*., 554–5.

20
*Ibid*., 558.

21 W. H. Willcox, ‘The Medico-Legal Importance of Wounds Produced by Firearms’, *Transactions of the Medico-Legal Society*, 1907–8, *5*, 5–20, 18. The comments made by members of the audience for papers read at the Medico-Legal Society are reproduced in the *Transactions*.

22 W. J. Bishop, ‘Willcox, Sir William Henry (1870–1941)’, rev. K. D. Watson, *Oxford Dictionary of National Biography* (Oxford, Oxford University Press, 2004) <http://www.oxforddnb.com/view/article/36910>, accessed 4 December. 2015.

23 Willcox, ‘Wounds Produced by Firearms’, 17–18.

24
*Ibid*., 8.

25 Sydney A. Smith, *Forensic Medicine: A Textbook for Students and Practitioners* (London: J. & A. Churchill, 1925), 129.

26
*Ibid*.

27 Sydney A. Smith, ‘The Investigation of Firearm Injuries’, *Transactions of the Medico-Legal Society*, 1928–29, *23*, 81–106, 104.

28 Susan Lindee, ‘Experimental Wounds: Science and Violence in Mid-Century America’, *Journal of Law, Medicine & Ethics*, 2011, *39*, 8–20; Susan E. Lederer, ‘Going for the Burn: Medical Preparedness in Early Cold War America’, *Journal of Law, Medicine & Ethics*, 2011, *39*, 48–53; Ian Burney, ‘War on Fear: Solly Zuckerman and Civilian Nerve in the Second World War’, *History of the Human Sciences*, 2012, *25*, 49–72.

29 Precognition of Thomas Middlemiss, Precognition against John Donald Merrett, 1927, Crown Office Precognitions (20th Century) National Records of Scotland, AD15/27/1 (Box 1 of 2), 13.

30 Roughhead, *Trial of John Donald Merrett*, 5–10, 20–3.

31 Transcript from the trial of John Donald Merrett for the crime of murder and uttering, Trial Transcripts, National Records of Scotland, JC36/53 (henceforth Merrett Trial Transcript), 104–5. Although photographs were often taken at Littlejohn’s autopsies, none from that on Mrs Merrett appear to survive. These photographs appear to have been kept for teaching purposes, rather than for use in court. Post-mortem photographs of Sir Henry Harvey Littlejohn, Department of Forensic Medicine, Records of the University of Edinburgh, Edinburgh University Library, EUA IN1/ACU/F1/3.

32 Merrett Trial Transcript, 606–7.

33 Henry Harvey Littlejohn, ‘Department of Forensic Medicine University of Edinburgh’, *Methods and Problems of Medical Education*, 1928, *9*, 187–99; John Glaister, Sr., ‘Forensic Medicine Department University of Glasgow’, *Methods and Problems of Medical Education*, 1928, *9*, 201–11.

34 The reason for this gap is unclear.

35 Medical report by John Glaister, 10 December 1926, Trial papers relating to John Donald Merrett, High Court of Justiciary Processes, National Records of Scotland, JC26/1927/27 (henceforth Glaister Medical Report, 10 December 1926); Medical report by Harvey Littlejohn, 13 January 1927, Trial papers relating to John Donald Merrett, High Court of Justiciary Processes, National Records of Scotland, JC26/1927/27 (henceforth Littlejohn Medical Report, 13 January 1927).

36 Smith, *Forensic Medicine*, 132.

37 Medical report by Harvey Littlejohn MB, 5 April 1926, Trial papers relating to John Donald Merrett, High Court of Justiciary Processes, National Records of Scotland, JC26/1927/27 (henceforth Littlejohn Medical Report, 5 April 1926); Merrett Trial Transcript, 104–5.

38 Littlejohn Medical Report, 5 April 1926.

39 Glaister Medical Report, 10 December 1926; Littlejohn Medical Report, 13 January 1927.

40 Crowther and White, *On Soul and Conscience*, 60–1.

41 Ian A. Burney and Neil Pemberton, ‘Bruised Witness: Bernard Spilsbury and the Performance of Early Twentieth-Century English Forensic Pathology’, *Medical History*, 2011, *55*, 41–60; Macdonald Hastings, *The Other Mr Churchill: A Lifetime of Shooting and Murder* (London: Harrap, 1963), 64.

42 Merrett Trial Transcript, 560–3, 587–99.

43 Cole, *Suspect Identities*, 183–4.

44 Golan, *Laws of Men and Laws of Nature*, 66–7.

45 Copy, letter from Glaister to Edinburgh Procurator Fiscal, 10 December 1926, Records of the Department of Forensic Medicine and Science, University of Glasgow Archive, FM/2B/20. Although the court records do not state whether the skin had been flayed from the leg before being experimented upon, this is unlikely, since Glaister, wrote later that experiments were made on ‘an adult lower limb which had been surgically removed from an injured man’, rather than upon skin therefrom. John Glaister, *A Text-Book of Medical Jurisprudence and Toxicology*, 5th edn (Edinburgh: E. & S. Livingstone, 1931), 316.

46 Merrett Trial Transcript, 560.

47
*Ibid*., 562.

48
*Ibid*., 297–8.

49
*Ibid*., 299–300.

50
*Ibid*.

51
*Ibid*., 594–5.

52
*Ibid*., 587.

53
*Ibid*., 594.

54
*Ibid*., 577.

55
*Ibid*., 306.

56
*Ibid*., 576.

57
*Ibid*., 605.

58
*Ibid*., 605–6.

59 John Glaister, Sr., *A Text-Book of Medical Jurisprudence and Toxicology*, 4th edn (Edinburgh: E. & S. Livingstone, 1921), 37–8.

60 Merrett Trial Transcript, 298. Littlejohn had written the foreword to Smith’s book.

61 Hastings, *The Other Mr Churchill*, 117–18.

62 Crowther and White, *On Soul and Conscience*, 83.

63 Regulations to be observed in criminal and other investigations, 1920, National Records of Scotland, AD5/14, 22, 141.

64
*Ibid*., 8.

65 Merrett Trial Transcript, 274.

66
*Ibid*., 579–80.

67
*Ibid*., 557.

68 Clark and Ambage, ‘Unbuilt Bloomsbury’, 298–9.

69 Joseph J. Levin, ‘A Case of Wounding by a Firearm’, *Transactions of the Medico-Legal Society*, 1925–26, *20*, 16–26, 24.

70 Smith, ‘The Investigation of Firearm Injuries’, 105.

71 Douglas J. A. Kerr, *Forensic Medicine: A Text-Book for Students and a Guide for the Practitioner* (London: A & C Black, 1936), 116–17.

72 A. Lucas, ‘The Examination of Firearms and Projectiles in Forensics Cases’, *Analyst*, 1923, *48*, 203–10, 210.

73 A. Lucas, *Forensic Chemistry* (London: Edward Arnold, 1921), 163.

74
*Ibid*., v–vi.

75 Sydney A. Smith, ‘The Identification of Firearms and Projectiles: As Illustrated by the Case of the Murder of Sir Lee Stack Pasha’, *British Medical Journal*, 2 January 1926, *1*, 8–10.

76 Smith, *Forensic Medicine*, 128–45, 464–6.

77 B. Kraft, ‘Critical Review of Forensic Ballistics: Part II’, *American Journal of Police Science*, 1931, *2*, 125–42, 142.

78 Ambage and Clark, ‘Unbuilt Bloomsbury’, 293.

79 Merrett Trial Transcript, 265–8.

80
*Ibid*., 576.

81
*Ibid.*, 562.

82
*Ibid*.

83 Precognition of Robert Churchill, Precognition against John Donald Merrett, 1927, Crown Office Precognitions (20th Century) National Records of Scotland, AD15/27/1 (box 2 of 2).

84 Merrett Trial Transcript, 284–5.

85
*Ibid*., 266.

86
*Ibid*., 594.

87
*Ibid*., 577–8.

88
*Ibid*., 266.

89 ‘Doctors Disagree’, *Scotsman*, 18 September 1931, 11.

90 ‘Position of the Gun’, *Scotsman*, 27 October 1931, 6.

91 ‘Perthshire Murder Trial’, *Scotsman*, 29 November 1935, 8.

92 ‘Perth Murder Charge’, *Scotsman*, 30 November 1935, 18.

93 Copy of report by [AM] and [JG] (First joint report), 29 November 1941, Records of the Department of Forensic Medicine and Science, University of Glasgow Archive, FM/2A/12J. Anonymised according to archive data protection regulations.

94 Sydney A. Smith and John Glaister, Jr., *Recent Advances in Forensic Medicine* (London: J & A Churchill, 1931), v, 1.

95 Crowther and White, *On Soul and Conscience*, 94–5.

96 Cole, ‘Witnessing Identification’.

97 Bates, ‘“So Far as I Can Define without a Microscopical Examination”’.

98 Crowther and White, *On Soul and Conscience*, 95.

99 Sydney A. Smith, *Mostly Murder* (London: Harrap, 1959), 145.

